# The Foot Orthoses versus Hip eXercises (FOHX) trial for patellofemoral pain: a protocol for a randomized clinical trial to determine if foot mobility is associated with better outcomes from foot orthoses

**DOI:** 10.1186/s13047-017-0186-5

**Published:** 2017-01-25

**Authors:** Mark Matthews, Michael Skovdal Rathleff, Andrew Claus, Tom McPoil, Robert Nee, Kay Crossley, Jessica Kasza, Sanjoy Paul, Rebecca Mellor, Bill Vicenzino

**Affiliations:** 1 The University of Queensland, School of Health and Rehabilitation Sciences, Sports Injuries Rehabilitation and Prevention for Health research unit, CCRE Spine, Brisbane, Australia; 2 Research Unit for General Practice in Aalborg and Department of Clinical Medicine, Aalborg, Denmark; 3 SMI, Department of Health Science and Technology, Faculty of Medicine, Aalborg University, Aalborg, Denmark; 4 Department of Occupational Therapy and Physiotherapy, Aalborg University Hospital, Aalborg, Denmark; 5 School of Physical Therapy, Rueckert-Hartman College for Health Professions, Regis University, Denver, USA; 6 School of Physical Therapy, Pacific University, Hillsboro, USA; 7 La Trobe University, School of Allied Health, College of Science, Health and Engineering, Melbourne, Australia; 8 Department of Epidemiology and Preventive Medicine, Monash University, Clayton, Australia; 9 Clinical Trials and Biostatistics Centre, QIMR Berghofer Medical Research Institute, Brisbane, Australia

**Keywords:** Treatment effect modifier, Clinical prediction, Prognosis, Knee pain, Management

## Abstract

**Background:**

Patellofemoral pain (PFP) is a prevalent, often recalcitrant and multifactorial knee pain condition. One method to optimize treatment outcome is to tailor treatments to the patient’s presenting characteristics. Foot orthoses and hip exercises are two such treatments for PFP with proven efficacy yet target different ends of the lower limb with different proposed mechanisms of effect. These treatments have not been compared head-to-head, so there is a dearth of evidence for which to use clinically. Only foot orthoses have been explored for identifying patient characteristics that might predict a beneficial effect with either of these two treatments. Preliminary evidence suggests patients will do well with foot orthoses if they have a midfoot width in weight bearing that is ≥ 11 mm more than in non-weight bearing, but this has yet to be verified in a study that includes a comparator treatment and an adequate sample size. This trial will determine if: (i) hip exercises are more efficacious than foot orthoses, and (ii) greater midfoot width mobility will be associated with success with foot orthoses, when compared to hip exercises.

**Methods:**

Two hundred and twenty participants, aged 18–40 years, with a clinical diagnosis of PFP will be randomly allocated with a 1:1 ratio to receive foot orthoses or progressive resisted hip exercises, and stratified into two subgroups based on their presenting midfoot width mobility (*high mobility* defined as ≥11 mm). The primary outcome will be a 7-point Likert scale for global rating of change. All analyses will be conducted on an intention-to-treat basis using regression models.

**Discussion:**

This trial is designed to compare the efficacy of foot orthoses versus hip exercise, as well as to determine if high midfoot width mobility is associated with better outcomes with foot orthoses when compared to hip exercises. Results of this trial will assist clinicians in optimising the management of those with PFP by testing whether a simple measure of midfoot width mobility can help to determine which patients are most likely to benefit from foot orthoses.

**Trial registration:**

This trial is registered on the Australian New Zealand Clinical Trials Register (ACTRN12614000260628)

## Background

Patellofemoral pain (PFP) is a prevalent knee condition throughout the lifespan [1–4], with a propensity to become persistent [5, 6]. Patellofemoral pain classically presents as anterior knee pain aggravated by activities that load the patellofemoral joint, such as climbing or descending stairs, running, squatting or sitting for prolonged periods [7]. Diagnosis is based on the clinical presentation of PFP, in the absence of other pathologies that might manifest as anterior knee pain [7].

Patellofemoral pain is also a multifactorial condition with guidelines suggesting optimal treatment should confer early pain relief and be targeted to the individual [7, 8]. Physical and exercise interventions for PFP are often targeted at the foot, knee and hip joints, or combinations thereof, with combined interventions proving superior [9]. Combined interventions for PFP often involve both active (e.g., progressive resistance exercise) and passive (e.g., orthoses, manual therapy, tape) therapies applied to the knee as well as the foot, thigh and hip regions. Selecting a tailored treatment plan for an individual patient from this range of interventions will potentially enhance treatment outcomes and minimise exposing patients to non-essential treatments.

Clinical trials have shown that exercising the hip muscles or using foot orthoses are efficacious in managing PFP, [10–12] but no studies have compared which is superior. Kinematic data suggests that the position and movement of the femoral bone, which is largely governed by hip joint movement under control of hip muscles, is the main contributor to patellofemoral joint loads [13, 14]. Exercise of the hip muscles would then plausibly have more effect on PFP through reduction of patellofemoral joint load, when compared to interventions targeting the foot (e.g., foot orthoses). We propose undertaking a comparison between hip exercise and foot orthoses, as it will address a common point of contention regarding whether proximal or distal approaches to PFP are more beneficial [15].

The recommendation to target treatments to the individual [8] has not been researched. One method of matching treatments to individual patients is to identify patient characteristics that can predict success after a specific treatment, known as treatment effect modifiers [16]. There are currently no valid treatment effect modifiers for treatment of PFP, but preliminary data suggest that further investigation of midfoot width mobility is warranted. Two studies have reported that greater midfoot width mobility [17] (defined as a change of 11 mm or more moving from a weight bearing to non-weight bearing posture) was present in greater proportions of participants reporting improvement in their condition when treated with foot orthoses [11, 18]. These preliminary studies are limited in terms of the methods required to prove treatment effect modification [19]. Such limitations include failure to compare the specific intervention of interest against another relevant treatment and testing too many potential predictor variables for the sample size studied.

We will undertake a randomized clinical trial that will investigate the role of midfoot width mobility as a treatment effect modifier for treatment of PFP with foot orthoses. It will also evaluate the clinical efficacy of foot orthoses against progressive resisted hip exercises. The prospective trial will stratify participants based on their midfoot width mobility and randomly allocate them to be treated with foot orthoses or hip exercises.

The objective of this trial is to determine if those individuals with PFP and greater midfoot width mobility will report better outcomes from foot orthoses when compared to hip exercises. The trial will also conduct a direct comparison between foot orthoses and hip exercises in the treatment of PFP.

Hypotheses:

(i) High midfoot width mobility is a treatment effect modifier for foot orthoses compared to progressive resisted hip exercises at 12 weeks. This means that beneficial effects of foot orthoses compared to hip exercises will be greater for patients with PFP who have high midfoot width mobility than in those who have low midfoot width mobility.
(ii) Hip exercises will be associated with better outcomes after 12 weeks, when compared to treatment with foot orthoses

## Method

This study protocol follows the Standard Protocol Items: Recommendations for Interventional Trials (SPIRIT) guidelines [20]. The study report will follow the CONSORT guidelines for randomized trials [21] with the extension for non-pharmacological treatments and TIDieR for intervention description [22, 23].

### Trial design

A two-arm prospective randomised superiority clinical trial in a multicentre setting with stratification on midfoot width mobility will evaluate if midfoot width mobility is a treatment effect modifier for foot orthoses compared to progressive resisted hip exercises. An independent off-site body will generate a randomisation schedule for all participants for both trial sites. Participants will be allocated into either foot orthoses or progressive resisted hip exercises in a 1: 1 ratio using permuted block randomisation stratified by site and by the mid foot mobility measure. The primary end point will be 12 weeks.

### Study setting

The trial will be conducted in Brisbane, Australia and Aalborg, Denmark. To reflect the common treatment settings in these countries, participants in Brisbane will attend private physiotherapy practices in the community while those is Aalborg will attend physiotherapy sessions in a hospital musculoskeletal outpatient department [24].

### Eligibility criteria

Volunteers will range from 18 – 40 years of age, report a history of anterior, retro or peri-patellar knee pain of non-traumatic origin that has persisted for more than six weeks. Self-reported worst pain over the previous week will be required to be greater than 3/10 on a numerical pain scale (0 = no pain, 10 = worst pain imaginable) with symptoms provoked by at least two or more of the following activities: squatting, running, prolonged sitting, stair ascending or descending. On physical examination, pain should be provoked by clinical palpation of the patellar borders, stepping down from a 25 cm step, during a double-leg squat and present on clinical compression of the patella into the trochlear groove. Eligible participants will be required to have basic comprehension of written and spoken English (Brisbane, Australia) or Danish (Aalborg, Denmark) because of the descriptive nature of pain and behavioral outcome measures applied in this study.

Volunteers will be excluded if they have any of the following: concomitant injuries or pathologies affecting other knee structures (e.g. ligament, meniscal, tendon, iliotibial band, pes anserinus), a history of knee or other significant lower limb surgery, patellofemoral dislocation or subluxation, Osgood-Schlatter’s disease, Siding-Larsen-Johanssen syndrome, a positive patellar apprehension test or evidence of knee joint effusion. Volunteers will be excluded if they present with any foot condition that may preclude the use of foot orthoses, pain in and/or referred from the hip, pelvis or lumbar spine, current use of anti-inflammatory or corticosteroid medication including injections, or any previous treatment for PFP or other conditions that included hip exercises or foot orthoses.

### Stratification criterion

An investigator at each trial site, different to the investigator responsible for enrolment, baseline and follow up outcome measures and blind to those outcome measures, will measure each participant’s midfoot width prior to treatment allocation. Midfoot width mobility is calculated as the difference in midfoot width between weight bearing and non-weight bearing postures and shown to be reliable [17]. The investigators taking the midfoot width mobility measurement will be trained to ensure they can reliably measure midfoot mobility. To test for midfoot width mobility as a treatment effect modifier for foot orthoses, we determined prior to the study that the stratification cutoff for midfoot width mobility will be 11 mm [11, 18]. Those who present with ≥11 mm midfoot width mobility will be defined as being *‘high mobility’* and those with <11 mm as *‘low mobility’*.

#### Interventions

Eligible participants will be randomly assigned to one of two interventions; (a) foot orthoses intervention or (b) a progressive resisted hip exercise intervention. Registered/licenced physiotherapists who regularly treat musculoskeletal conditions will deliver both interventions. Treating physiotherapists at both sites will be trained by the same investigators (BV, MM & MSR) in the intervention protocols for both foot orthoses fitting and hip exercises prior to trial commencement to ensure consistent implementation of the interventions. Although the treatments are standard physiotherapy interventions, to ensure fidelity of treatment application all clinicians will be provided with extensive documentation including images of treatments, have an option to attend a refresher workshop, and access to a senior investigator for any queries or issues that arise during the trial. Treating physiotherapists will be blind to the participant’s midfoot width mobility measurements and baseline and follow-up outcome measurements. At the start of the study all participants will receive education to facilitate a basic understanding of their PFP condition and advice on physical activity. Participants will be encouraged to remain physically active provided that their chosen activities do not provoke pain that persists after ceasing their activities, and there is no general deterioration of symptoms during or after the cessation of activity.

### Foot orthoses

**Fig. 1:**
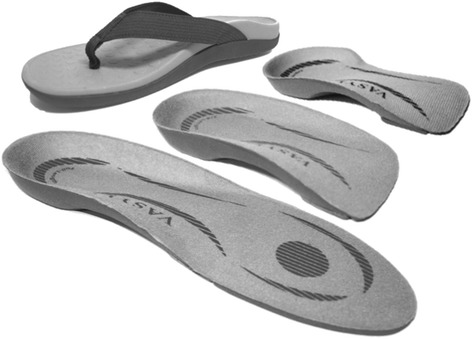
Orthoses types. (*From front*) Full length, three-quarter length, easy fit & contoured sandal

**Fig. 2:**
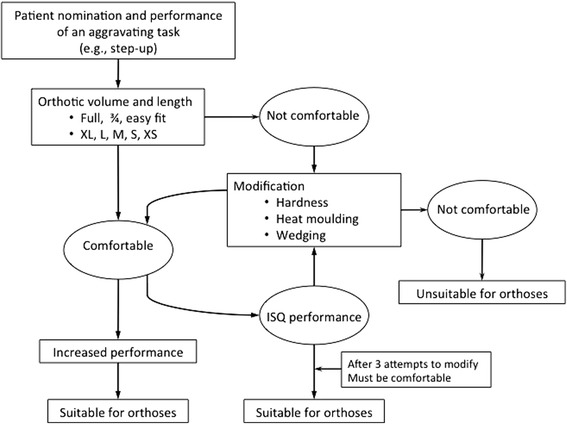
Flowchart of orthoses fitting procedure

Prescription of foot orthoses will follow the protocol utilised in a previous randomised control trial [10]. Physiotherapists will be provided with a range of commercially available prefabricated foot orthoses (Vasyli International, Labrador, Australia) (Fig. 1). The orthoses are manufactured and designed from ethylene-vinyl acetate with an inbuilt arch support and a manufacturer specified 6° varus wedge. The orthoses are constructed in 3 different levels of hardness [high (Shore A 75°), medium (Shore A 60°) or low (Shore A 52°)]. Prior to fitting the orthoses, the participant will perform a nominated aggravating task (e.g., step-ups). Physiotherapists will then follow a standardised fitting procedure (Fig. 2). The physiotherapist has the scope within the fitting procedure to review the size, length, and hardness of the orthoses, that prioritises comfort as this is a key determinant of participant compliance [25]. To maximise comfort of the orthoses, physiotherapists can make modifications including heat moulding and/or trialing various medial wedges to the rear foot (2° or 4° inclination) and/or forefoot (4° or 6° inclination) and/or heel raise (4, 6 or 8 mm in height). Once the participant is satisfied with the comfort of the orthoses, the participant will perform the previously nominated aggravating task. An improved performance will be determined by the participant reporting a reduction in pain score or improved performance (e.g. more repetitions of an aggravating activity) before the onset of their pain.

The prescribing physiotherapist will have 3 attempts to modify the foot orthoses to primarily be comfortable and then improve performance of the participant selected task. In the unlikely event that the foot orthosis cannot be modified sufficiently to the participant’s satisfaction by the third session, then the participant will be deemed unsuitable for foot orthoses. That is, no participant will be asked to wear orthoses that they perceive is uncomfortable. Previous trials of the same population using the same fitting procedure reported that no participants were unsuitable for this intervention [10, 11].

To encourage wearing of the orthoses, participants will be prescribed up to four pairs to fit a wide range of footwear as well as contoured (in the form of the orthoses) sandals for everyday use. Participants will be encouraged to wear the orthosis or contoured sandal whenever weight bearing. The sandal and orthoses have been shown to similarly increase arch height in healthy participants [26].

**Fig. 3:**
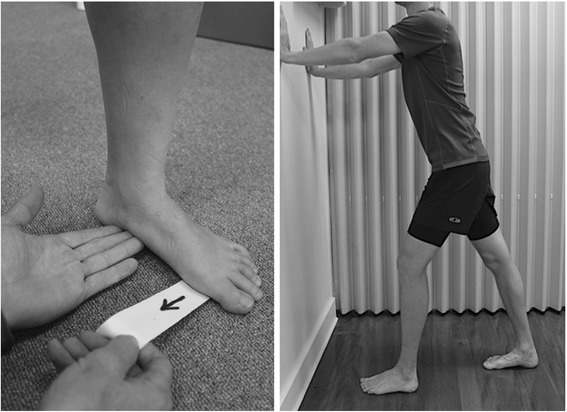
Foot and ankle exercises.(*Left*): Anti-pronation exercise: The rearfoot is supinated (with tactile feedback) whilst maintaining first metatarsal head in ground contact. The white non-elastic tape is placed under the distal first metatarsal and the participant asked to prevent it from being removed (i.e., through plantarflexion of the first ray) by the clinician who exerts traction on the tape. (*Right*) Calf stretch exercise, which is performed with the foot in netural position and the midline of the foot and the mid-point of the patella kept perpendicular to the wall

Participants receiving orthoses will also be asked to perform a home foot and ankle exercise program twice per day (Fig. 3). The program will include (i) stretches for the triceps surae/tendo-Achilles complex (3 × 30 sec weight-bearing), and (ii) anti-pronation postural foot exercises. The anti-pronation foot exercises aim to improve the participant’s awareness from a relaxed pronated posture to a more supinated posture. Therapists will initiate training of the foot exercises with participants seated with the knees flexed and bare feet on the ground. Training consists of verbal and manual facilitation of participants to supinate the rear foot (manual facilitation: therapist upward pressure under the navicular as well as palpating the talocrural joint space for medio-lateral symmetry), while maintaining the first metatarsal head firmly on the floor and the toes relaxed. This foot posture will be held for 5 × 10 s. The exercises will be performed on each foot separately. Participants will attend a total of six sessions over six weeks.

### Hip exercises

The progressive resisted hip exercise protocol is modified from a protocol successfully used to improve outcomes at 12 months in women with PFP [27]. Exercise therapy focused on hip muscle groups, in particular hip abductor, external rotator, and extensor muscle groups, as well as a knee strengthening and stretching program targeting quadriceps, hamstrings and triceps surae muscle groups [27]. Results from intervention studies [12, 27–31] support that exercises targeting the postero-lateral hip musculature can improve long-term function and reduce PFP when compared to no exercises or knee exercises alone [32].

**Fig. 4:**
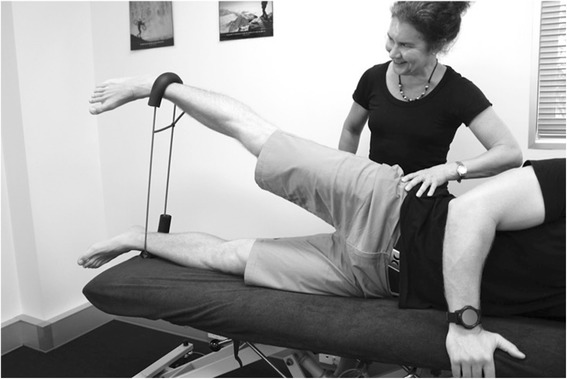
Hip abduction exercise in side lying

**Fig. 5:**
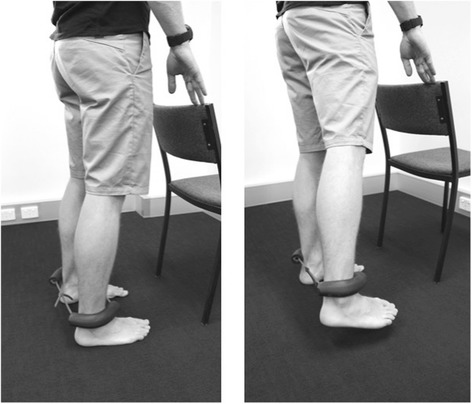
Hip abduction exercise in standing

**Fig. 6:**
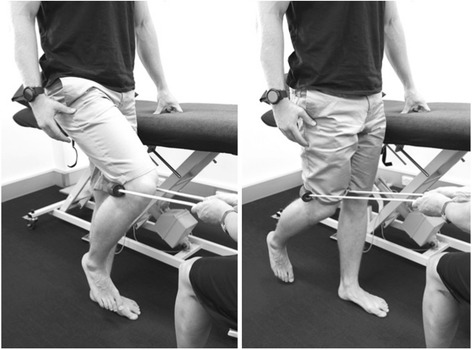
Hip extension exercise in standing

**Fig. 7:**
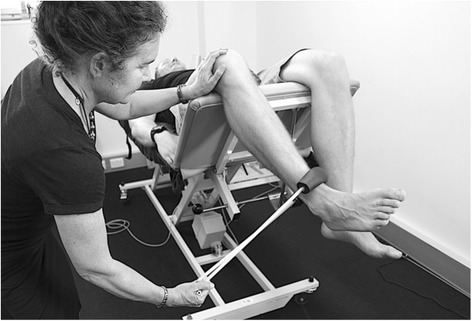
Hip external rotation exercise in supine and with the hip in 30° flexion

Participants in the progressive resisted hip exercise group will attend three sessions per week for four weeks (12 sessions) [27] to perform exercises focused on the hip abductor (Figs. 4 and 5), extensor (Fig. 6) and external rotator (Fig. 7) muscles groups. The exercises will be performed alternately on both sides and are described in Table 1 [33]. Elastic bands will provide resistance for the exercises and will be standardized to allow the participant to achieve a maximum of 10 repetitions. Resistance (denoted by band colour) and length (50, 60, 70 cm loops) of the band (Theraband™) will be selected by the physiotherapist to suit individual participant capacity, re-evaluated at each treatment session and progressed accordingly. Using an 11-point scale of perceived exertion, participants will be encouraged to exercise at a rate of 5–7 (‘Hard’ to ‘very hard’) (Table 2). The contraction phase for each repetition will be 2 s concentric, 1 s isometric, 2 s eccentric and 1 s rest; with approximately a 90 s rest between each set of 10 repetitions, while training the contralateral side.

**Table 1:** Hip exercise descriptors

	Hip abduction (side lying) (Fig. 4)	Hip abduction (standing) (Fig. 5)	Hip extension (Fig. 6)	Hip external rotation (Fig. 7)
Load magnitude	Approximately 10-12RM	Approximately 10-12RM	Approximately 10-12RM	Approximately 10-12RM
Number of repetitions	10	10	10	10
Number of sets	3	3	3	3
Rest in-between set (s)	Approx. 90s	Approx. 90s	Approx. 90s	Approx. 90s
Number of exercise interventions (per week)	3/week	3/week	3/week	3/week
Duration of the experimental period (weeks)	4 weeks	4 weeks	4 weeks	4 weeks
Fractional and temporal distribution of the contraction modes per repetition and duration (s) of one repetition	2 s concentric 1 s isometric 2 s eccentric	2 s concentric 1 s isometric 2 s eccentric	2 s concentric 1 s isometric 2 s eccentric	2 s concentric 1 s isometric 2 s eccentric
Rest in-between repetitions (s)	1 s	1 s	1 s	1 s
Time under tension (s)	5 s/rep 50s/set 150 s/exercise session 1800s/total intervention	5 s/rep 50s/set 150 s/exercise session 1800s/total intervention	5 s/rep 50s/set 150 s/exercise session 1800s/total intervention	5 s/rep 50s/set 150 s/exercise session 1800s/total intervention
Volitional muscular failure	No	No	No	No
Perceived exertion (/11) (Table 2)	5-7/11 (‘Hard’ to ‘very hard’)	5-7/11 (‘Hard’ to ‘very hard’)	5-7/11 (‘Hard’ to ‘very hard’)	5-7/11 (‘Hard’ to ‘very hard’)
Range of motion (degrees)	0° to approx. 30°	0° to approx. 30°	45° hip flexion to approx 0°	0° to approx. half of available external rotation range°
Recovery time in-between exercise sessions ((hr)	48 hr	48 hr	48 hr	48 hr
Anatomical definition of the exercise (exercise form)	Side lying with the symptomatic leg top-most. Elastic band is placed around the ankle of the symptomatic leg and attached to the end of plinth. Participants abduct the leg up to 30° hip abduction and return back from the bed.	The participant will stand with the elastic band looped around both ankles, superior to lateral malleoli. Prior to the exercise, the target hip will be in slight internal rotation (to minimize incorrect compensatory action of external rotation during abduction). Hip abduction will then be performed to approximately 45°.	The participant will stand with target hip in 45°hip flexion. One end of the elastic band fixated (or held by the therapist) at knee height and looped around the back of the knee. The hip is then extended whilst maintaining a neutral lumbo-pelvic position.	With the participant supine, and hips in 30° flexion over a wedge. Elastic band is placed around the ankle of the symptomatic leg and held by the therapist. Participants externally rotate the hip against resistance to mid-range of available external rotation.

**Table 2:** Borg scale of perceived exertion

1-10 Borg Scale of Perceived Exertion	
0	Rest
1	Really Easy
2	Easy
3	Moderate
4	Sort of Hard
5	Hard
6	
7	Really Hard
8	
9	Really, Really Hard
10	Maximal

For each of the twelve sessions, the treating physiotherapist will record attendance, strength (colour) and length of band used for each exercise, number of sets and repetitions completed as well as any adverse effects. At the completion of the program, participants will be instructed to continue with normal activities of daily living with no instructions to continue on with a home exercise program.

### Outcome measures

The outcome measures will be a range of self-reported questionnaires, including psychological and quality of life measures as these are often involved in persistent musculoskeletal pain conditions, and functional tasks that load the patellofemoral joint. Participants will not be made aware of the specific study aim to evaluate midfoot width mobility as a treatment effect modifier so as to minimise the impact of participant expectation of treatment response on the basis of their foot type (or their allocated treatment group). Baseline and follow up (6 and 12 weeks after the commencement of intervention) outcome measures will be administered by an assessor at each trial site who will be blind to the participant’s midfoot width mobility measurement and intervention allocation.

#### Primary outcome measurement (6 and 12 weeks)

The global rate of change scale (GROC) is the primary outcome measure with the primary endpoint at 12 weeks. The GROC is a participant rating of the direction and magnitude of overall change in symptoms [34]. Participants will be asked: “How would you describe your knee pain now, compared to before you began the treatment.” They will answer this question by selecting a descriptor on a 7-point Likert scale that best represents any change in their symptoms (much better, better, a little better, no change, a little worse, worse, much worse). Global rating of change scales have been frequently used in studies investigating treatment outcome in those with PFP and shown to be a flexible, simple and sensitive method for measuring meaningful individual improvement [10, 11, 35–37]. For analysis purposes, the GROC will be dichotomized so that ‘much better’ and ‘better’ represent success with treatment.

#### Secondary outcome measures

Single assessment numeric evaluation (SANE): Single assessment numeric evaluation questions have been used previously in participants with neck pain [38], shoulder surgery [39] and anterior cruciate ligament reconstruction [40] and been shown to correlate well with other outcome measures. Participants will be asked:

(i) “How would you rate your knee today as a percentage of normal on a scale of 0% to 100%?” with 100% being defined as having no problems at all with the knee. (at 0, 6 and 12 weeks)
(ii) “On a scale of 0 (not at all) to 100% (totally recovered), how well do you feel you have recovered from your knee pain?” (at 6 and 12 weeks)

Patient acceptable symptom state (6 and 12 weeks): Patient acceptable symptom state is defined as the highest level of symptom beyond which patients consider themselves well [41]. Patient acceptable symptom state has been used in musculoskeletal and rheumatic conditions and shown to provide information about a patient’s improvement exceeding the minimally clinically important improvement [41–43]. Participants will be asked to answer yes or no to a structured question: “Is your current condition satisfactory, when you take your general functioning and your current pain into consideration?”

Perception of success and willingness to recommend the treatment (6 and 12 weeks): Participants will be asked to answer yes or no to two questions in regards to their perception of the success of their treatment:

(i) “Overall, would you agree that the treatment you have received has been successful for your knee pain?”
(ii) “If a good friend has the same knee pain as you, would you recommend the same treatment you received?”

Patient satisfaction (6 and 12 weeks): Participants will be asked two questions in regards to the satisfaction of their treatment with a selection of five possible responses (very satisfied, somewhat satisfied, neither satisfied not dissatisfied, somewhat dissatisfied, very dissatisfied). The questions will be:

(i) “Over the course of treatment for your knee pain, how satisfied were you with your overall treatment?”
(ii) “If you had to live with the symptoms you have right now, how would you feel about it?”

Numerical pain rating scale (0, 6 and 12 weeks): The numerical pain rating scale (NPS) can be a verbal or visual scale to grade the intensity of pain experienced by the participant and is recommended for research purposes [44]. Participants will be asked to indicate a score that best represents the intensity of their knee pain on an 11-point scale where 0 represents no pain and 10 represents worst pain imaginable. Participants will provide two ratings; their average pain over the previous seven days and their worst pain over the previous seven days. An improvement of ≥ 2 on the NPS indicates clinically meaningful change [45, 46].

Patient specific functional scale (0, 6 and 12 weeks): Participants will self-select up to five tasks or activities that are impaired due to their symptoms. Participants will then rate the level of impairment of each task/activity on an 11-point scale from 0 (“unable to perform activity”) to 10 (“able to perform activity at same level as before the injury or problem”). The patient specific functional scale is a reliable and valid tool that is sensitive to changes in patient’s symptoms [47, 48]. It has been reported that a change of three or more on an individual patient-nominated activity indicates a true change in functional capacity [48].

Kujala Patellofemoral Scale (0, 6 and 12 weeks): This questionnaire comprises 13 items designed specifically for PFP. Categories within the questionnaire cover a range of knee functions under varying loads. Participants select a response to each of the 13 items that best depicts their symptoms. Each item is weighted separately and then summed overall, with the highest possible score of 100 points representing pain free full function and 0 representing total incapacity. This questionnaire has been recommended for knee pain because it is reliable and sensitive to changes in symptoms [49–51]. A change of 10 points is considered as the minimum clinically important difference [50] in patients with PFP.

Knee injury and osteoarthritis outcome scale (KOOS) (0, 6 and 12 weeks): This questionnaire is comprised of five separate subscales that assess the patient’s opinion of their knee and symptoms. The subscales cover pain, symptoms, activities of daily living function, sporting and recreation function and quality of life. Each subscale consists of standardized answers (five Likert boxes), with each question scored 0–4 separately. The questionnaire will be scored according to the 2012 KOOS scoring manual. Participants select a response to each question in each subscale that best depicts their symptoms. Each subscale will be normalised to a scale of 0–100 (0 = extreme problems, 100 = no problems). A change of 8–10 points is suggested to represent a clinically significant change in symptoms [52].

Hospital anxiety and depression scale (HADS) (0, 6 and 12 weeks): This 14-item scale will be used to investigate emotional states of those with PFP. It has been found to be a reliable instrument for detection of anxiety and depression in an outpatient setting and a valid indicator of severity [53, 54]. Participants are required to select the best of four responses to questions pertaining to either anxiety or depression (seven questions each), which are scored from 0 to 3. The scores for the anxiety and depression questions are summed separately to give total scores for each component, where 0–7 represents no anxiety or depression, 8–10 is borderline, and 11–21 indicates the presence of an anxious or depressive state.

Euro-Qol™ (EQ-5D 3 L version) (0, 6 and 12 weeks): This validated questionnaire is used as a measure of health outcome and provides a simple descriptive profile and a single index value for health status. It compromises of five domains about mobility, usual activities, self care, pain and discomfort, and anxiety or depression [55]. Participants will be asked to rate their impairment on each domain (none, moderate or severe problems). Each health state is scored (1–3) and transformed into an index score. This score is used to derive quality-adjusted life years as an outcome measure and is one of the most commonly used economic evaluations used to inform decisions in health care [56]. The participant scores their overall health on a 0 to 100 scale, where 100 represents complete health and well-being [55].

Tampa scale for kinesophobia (TSK) (0, 6 and 12 weeks): The TSK is a 17-item questionnaire aimed at assessing fear of reinjury due to physical movement [57]. Each item is scored on a 4-point Likert scale that ranges from strongly disagree (1) to strongly agree (4). The inverse scores from items 4, 8, 12, and 16 are used to calculate the total score. Total TSK scores range between 17 and 68, with higher scores suggestive of higher levels of fear of physical movement and vulnerability.

Pain Catastrophising Scale (PCS) (0, 6 and 12 weeks): The PCS is a 13-item valid and reliable questionnaire that evaluates a participant’s level of pain catastrophic thinking, and classifying this into levels of rumination, magnification and helplessness [58]. Participants are asked to reflect on past painful experiences, and to indicate the degree to which they experienced certain thoughts or feelings when experiencing pain, on 5-point scales from not at all (0) to all the time (4). The PCS yields a total score and three subscale scores for rumination, magnification and helplessness respectively. The total score ranges from 0 – 52, with higher scores indicating higher levels of pain catastrophization.

Functional tests: Step down, step up and squat (0, 6 and 12 weeks): These functional tests are commonly reported as aggravating activities by patients with PFP because they load the patellofemoral joint and have been previously used in clinical trials [10]. Repeated step testing will be performed on a single 25 cm step in time with a metronome set at 96 beats per minute (e.g., stepping up/down on each beat). Repeated squats will be performed in time with a metronome set to 96 beats per minutes feet shoulder width apart, squatting down in two beats, until the participant can touch both lateral malleoli with their fingers, and standing up over two beats. Activities will be stopped when either a) onset of symptoms occurs, or b) there is an increase in existing symptoms or c) when a maximum of 25 repetitions has been reached without the onset of pain.

### Physical measurements

An examiner at each trial site, who is blinded to treatment allocation and midfoot width mobility stratification, will collect self-reported questionnaires (i.e., pain scores, Kujala Patellofemoral Scale, etc.), physical measurements and demographic data prior to commencement of the intervention and at follow-up. Physical measurements will include foot posture measurements, ankle, hip and first metatarsophalangeal range of motion measurements and maximal isometric hip strength testing. These measures will be used in post-hoc analyses of prognostication and identification of other possible candidates for treatment effect modifiers.

**Fig. 8:**
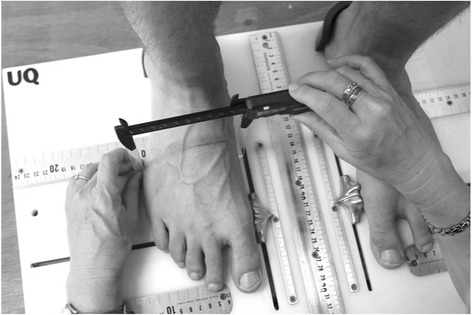
Midfoot width measured in weight bearing

**Fig. 9:**
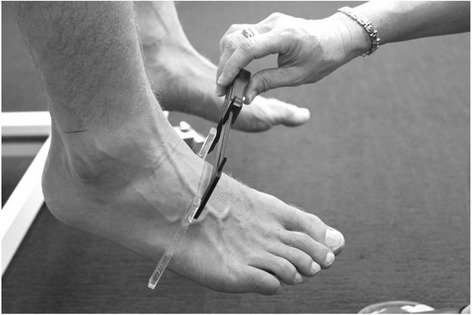
Midfoot width measured in non-weight bearing

**Fig. 10:**
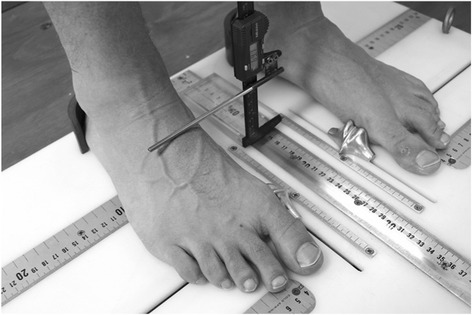
Midfoot arch height measurement

Midfoot width and height mobility: Measurement of the width and height at the midfoot (i.e. 50% of total foot length) has been previously described and demonstrated to be reliable [17]. In brief, these measurements are performed on a foot measurement platform that can standardize foot position by placing heels 15.24 cm apart with the first metatarsal heads against a guide with body weight equally distributed on both feet. Midfoot width in weight bearing is measured using a digital caliper with extend arms, which are positioned perpendicular to the sole of foot and adjacent to lateral and medial aspect of the foot at the 50% length (Fig. 8). This is repeated in non-weight bearing with the patient seated on a height adjustable table and legs hanging freely (Fig. 9). Midfoot height (dorsal arch height) measurements at 50% of the total foot length in weight bearing (bipedal stance) and minimal weight bearing postures will also be taken (Fig. 10) [17]. To measure the arch height in a minimal weight bearing posture, the participant sits on a height adjustable plinth with their feet hanging freely. The assessment platform is positioned under both feet and the plinth is lowered until the point of the heel being assessed just contacts the platform. The vertical height of the arch is then measured. The height and width measurements of each foot in weight bearing and non (or minimal) weight bearing will be recorded separately three times and then averaged to give a single value for the analysis. The change in midfoot height and width is calculated by subtracting the measures in the two weight bearing conditions.

Navicular drop: The participant stands barefoot with equal weight on both feet. The navicular tuberosity will be identified using palpation and the most prominent point marked using a water-soluble ink pen. With the patient standing in subtalar joint neutral position (defined by palpation of the talus in the mortise and scored ‘0’ on the foot posture index [59]), the height of the navicular tuberosity will be measured using a clear angle ruler. The participant is instructed to relax their feet and the navicular tuberosity height is re-measured. The difference in height measurements between a subtalar joint neutral and relaxed foot position will be calculated to determine the amount of navicular drop [60–63].

The Foot Posture Index (FPI-6): Relaxed foot posture will be assessed using the FPI-6, which consists of six criteria: (i) talar head palpation, (ii) curves above and below the lateral malleoli, (iii) inversion/eversion of the calcaneus, (iv) bulge in the region of the talonavicular joint, (v) congruence of the medial longitudinal arch and (vi) abduction/adduction of the forefoot on the rearfoot [59]. Each criterion is examined and scored on a 5-point scale between −2 and +2, which are then totaled to categorize the foot as being highly pronated, pronated, normal, supinated, or highly supinated [59]. Intrarater reliability has been reported to be very good with interrater reliability being only moderate between three raters [64, 65].

Weight bearing bent knee ankle dorsiflexion (Lunge Ankle Dorsiflexion Device - LAD): Bent knee ankle dorsiflexion will be measured using a bespoke device, the Lunge Ankle Dorsiflexion measurement device (LAD). The LAD device has been previously described [66]. In brief, the LAD was designed with only one degree of freedom of motion in the sagittal plane. The patient’s foot is aligned in a sagittal plane with a line that bisects the 2nd and 3rd phalanges and the midline of the posterior calcaneus. Whilst maintaining the toe in light contact with the front of the reference block, the participant slowly lunges forward, with the knee in contact with a mobile measurement indicator. The therapist focuses on ensuring that the three points remain in the sagittal plane by watching for heel drift (usually medially) and heel lift, which indicates that full dorsiflexion has been reached. The linear measurement of horizontal distance between anterior knee and the fixed reference block at the longest toe is read from a ruler (mm).

**Fig. 11:**
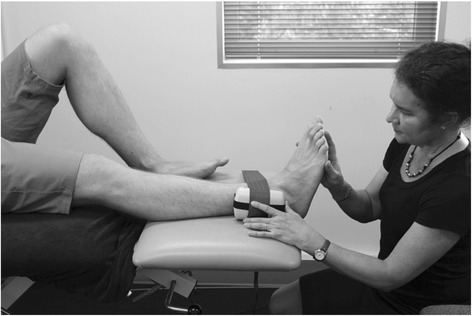
Hip abduction strength testing

**Fig. 12:**
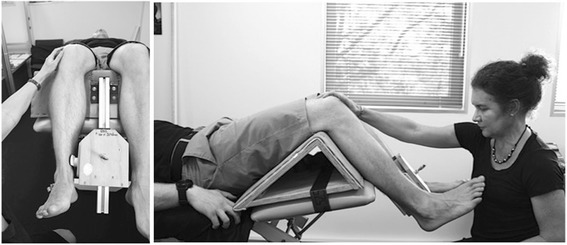
Hip external rotation strength testing in 30° hip flexion

Hip strength: Strength of the hip abductors, adductors and external rotators will be measured at baseline, 6 and 12 weeks as dysfunction in these muscle groups has been identified as a common impairment within the PFP population, [67–69] and will be used in post hoc exploratory prognostic analyses. Force produced during a maximal voluntary isometric contraction (MVIC) will be measured with a hand held dynamometer (Nicholas, Lafayette, IN47903, USA) Measurements will take place in supine to minimize the effect of gravity during testing and compensatory contractions [70]. Each participant will complete two practice contractions (50% MVIC followed by 100% MVIC) followed by three experimental MVICs where the participant will be asked to contract maximally for 5 s. Participants will have a 30 s rest between each contraction. The peak force (Newtons) will be recorded for each contraction and converted to torque (using the distance between the point or rotation and placement of dynamometer as the lever arm) standardized to body mass (Nm/kg). Hip abductor and hip adductor muscle strength will be tested using a dynamometer 5 cm proximal to the lateral and medial malleolus respectively, and stabilised by a rigid belt. The test leg will be extended in 0° abduction and 0° flexion, with the non-test hip and knee flexed (Fig. 11). Hip external rotation will be measured in supine with the hips in 30° of flexion with the dynamometer 5 cm proximal to the medial malleolus, stabilised in a solid bracket, fixated to the testing device (Fig. 12). This testing position was chosen because it: corresponds to biomechanical data on muscular actions of the external rotators in various degrees of hip flexion (i.e., piriformis being an external rotator muscle at 0° flexion and functionally switch rotation action to internal rotation at >60° hip flexion); [71, 72] replicates the position of exercise in the hip intervention protocol [27]; and approximates the degrees of hip flexion relative to the pelvis during foot contact/limb loading in the initial stance phase of gait [73–75].

Limb length for the hip abductor and adductor measurements, will be measured from the participant’s anterior superior iliac spine to a mark 5 cm proximal from the lateral and medial malleolus, respectively. For hip external rotation, distance will be measured from the medial joint line to a mark 5 cm proximal to the medial malleoli. Participants will be instructed to hold the sides of the plinth for stabilization and receive a standard verbal encouragement with consistent level of volume and enthusiasm.

**Fig. 13:**
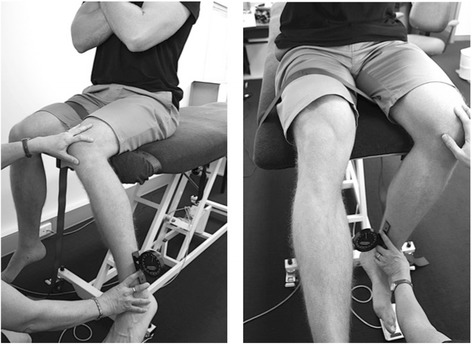
Hip internal and external range of motion measuring

Hip range of motion: Passive hip internal and external rotation range of motion will be measured in upright sitting, arms crossed, knees flexed to 90° over the edge of the plinth and the non-test leg stabilised by a rigid belt. The hip will be passively rotated to the point of resistance with no compensatory pelvic motion. Range will be measured using a plurimeter placed 5 cm proximal to the tip of the tibial malleoli on the medial border of the tibia for external rotation, and 5 cm proximal to the tip of the lateral malleoli to measure internal rotation (Fig. 13).

Demographic and other information: Other baseline measurements to be collected will include age, sex, height, weight, body mass index (BMI), unilateral and bilateral symptoms, duration of symptoms, use of medications, physical activity levels, joint mobility using the Beighton and Horan Joint Mobility Index [59, 64] and reported crepitus during daily living activities.

### Participant timeline

**Fig. 14:**
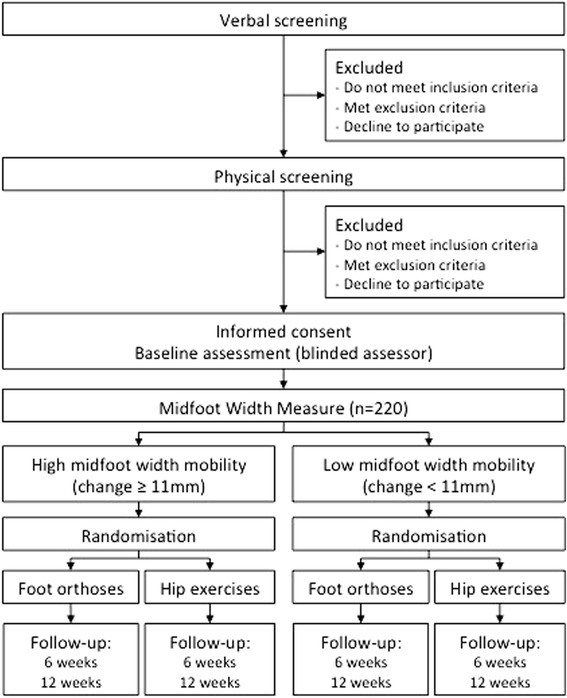
Flowchart of participants through trial (consort)

Volunteers will be recruited into the study through a structured process involving a comprehensive advertising campaign followed by verbal and physical examination screening of eligibility by a registered physiotherapist. Participants who meet the eligibility criteria will be offered enrolment into the study, complete consent forms then undergo baseline measurements and randomly allocated to an intervention (Fig. 14). Participants with bilateral symptoms will nominate their most symptomatic knee to be used in analysis. The timeline for events (e.g., outcome measure timepoints and close out) are shown in Table 3.

**Table 3:** SPIRIT figure. Schedule of enrolment, interventions and assessments

		Trial period								
	Enrolment	Allocation	Intervention period						Follow up	Close out
	May 2014– November 2016	May 2014– November 2016	Week 1	Week 2	Week 3	Week 4	Week 5	Week 6	Week 6	August 2014 – Feb 2017
Enrolment										
Eligibility screening	X									
Informed Consent	X									
Allocation		X								
Intervention										
Foot orthoses			X	X	X	X	X	X		
Hip exercises			X	X	X	X				
Assessment										
Diagnosis	X									
Midfoot width mobility	X									
Demographics		X								
Global rating of change									X	X
Rate of recovery									X	X
Patient acceptable symptom state									X	X
Numerical pain rating		X							X	X
PSFS		X							X	X
Kujala		X							X	X
KOOS		X							X	X
HADS		X							X	X
Euro-QoL		X							X	X
TSK		X							X	X
PCS		X							X	X
Functional tests: step up, step down, squat		X							X	X
Navicular height		X								X
Midfoot height mobility		X								X
Isometric hip strength testing		X							X	X
Range of motion measures		X							X	X

*Kujala* Kujala patellofemoral pain scale, *PSFS* patient specific functional scale, *KOOS* knee injury and osteoarthritis outcome scale, *HADS* hospital anxiety and depression scale, *TSK* Tampa scale for kinesophobia, *PCS* pain catastrophising scale

### Sample size

Sample size was based on proportions of patients rating themselves as “better” or “much better” on the Global Rating of Change (GROC) score in the foot orthoses and hip exercise treatment groups. The primary aim of the study is to determine whether midfoot width mobility is a treatment effect modifier for foot orthoses when compared to progressive resisted hip exercises. This requires testing for an interaction between midfoot width mobility, dichotomised as high (≥11 mm) or low (<11 mm), and treatment group. Based on previous findings, which indicated a strong effect of foot orthoses in patients with PFP who had a midfoot mobility ≥11 mm, [11] we wanted to be able to detect an interaction effect of 50 percentage points. This means that the difference between the foot orthoses and hip exercise groups in the proportions of participants who are improved at 12 weeks will be 50 percentage points higher (favoring foot orthoses) in participants with high midfoot width mobility than in those with low midfoot width mobility. A sample of 30 participants (15 per group) who have high midfoot width mobility provides 80% power using a two-sided significance level of 0.05 to detect a difference between the proportions of participants with improvement of 30% in the hip exercises group compared to 80% in the foot orthoses group. Assuming that 20% of participants will be in the high midfoot width mobility group, we inflated the sample size to 188 participants (94 per group) to ensure adequate power to detect this interaction effect of 50 percentage points [76]. To allow loss to follow-up of up to 15%, the final sample size was 220 participants (110 per group).

### Recruitment

A comprehensive recruitment strategy, successfully utilized in previous clinical trials [11, 18] will be used in regions of Brisbane, Australia and Aalborg, Denmark. The recruitment strategy involves paid advertisements in local and regional newspapers, supplemented by advertisements on university, gymnasium and community websites, online social media, electronic and paper noticeboards within the catchment area at regular intervals during the recruitment period. Further referrals may come from physiotherapists involved in the study and general practitioners, through the provision of information and advertising packages at their practices. Volunteers who express interest in participating will be screened through the previously described two-stage screening process to determine eligibility.

### Allocation

Once informed consent and baseline measurements have been obtained, each participant will be randomly allocated to one of two intervention groups via concealed allocation and assigned a participant code. An independent off-site body will generate a randomization schedule for all participants at both the Australian and Danish sites. The randomization schedule will be generated by computer and allocate on a 1:1 basis to each of the treatments with stratification on the midfoot width mobility measure.

### Data collection and management

All data will be collected in paper format and subsequently entered into an electronic study database. A number of strategies have been employed to ensure fidelity of data entry, such as entries will be screened at random by a second investigator to ensure entry is correct. The study database has been developed in a regulatory approved electronic medical records platform (OpenClinica®) by the Clinical Trials and Biostatistics Unit. This database will be used to comprehensively collect all safety and efficacy related data, along with additional information for possible exploratory analyses. The database development, testing, validation and management strictly follow the regulatory guidelines for clinical trial data management. All participant data will be analysed on an intention-to-treat basis. Once a participant is enrolled, every reasonable effort will be made through paper and electronic media to maintain contact and follow the participant for the duration of the trial period. It is anticipated that the rate of loss-to-follow-up will be at most 10%. Participants will be informed they may withdraw from the study at any time, for any reason without any consequences. Participants may be withdrawn from the study in order to protect their safety (e.g., the foot orthoses intervention is unable to be made comfortable to wear) after consulting with the senior investigator (BV).

### Statistical methods

A biostatistician who is blind to treatment group allocation and midfoot width mobility will conduct analysis. All participants who have missing data and did not fully comply with the treatment protocol will be included in analyses. Demographic characteristics will be inspected to assess baseline comparability of treatment groups and compare those participants who remain in the study and those who withdraw. If the proportion of missing data for endpoints exceeds 5%, multiple imputation methodology will be applied. To test the hypothesis of interaction between randomised group and foot mobility, terms for randomised group and foot mobility group, together with an interaction between the two, will be included in models. For the primary outcome (dichotomised GROC) and other binary secondary outcomes, binary regression models with a logarithmic link will be fit. For other outcomes, linear regression models will be fitted, and assumptions will be assessed using standard diagnostic plots. To test for an overall treatment effect, regression models for outcomes will include terms for randomised group and foot mobility (as foot mobility is a stratifying variable).

We will also undertake a secondary analysis to further explore the relationship between midfoot width mobility and the outcome, whereby midfoot width mobility will be included in the model as a continuous variable, together with an interaction term with randomised group. Relationships will be investigated using fractional polynomials [77]. We elected to perform this secondary analysis because previous studies that identified midfoot width mobility as a potential predictor of outcome after foot orthoses used data-dependent techniques in relatively small samples to establish a cut-off value for “high” midfoot width mobility. The concern with establishing cut-off values with data-dependent techniques is that, while the cut-off value may have been “optimal” for the original sample, this same cut-off value may not be optimal in the larger population [78, 79].

### Monitoring

A safety committee will be established when the need arises. It is not anticipated that a safety committee will need to convene much or at all, because the treatments have been previously studied with no reported serious adverse events, are common to everyday practice for this condition and there is low perceived risk to participants. Participants and the treating physiotherapists are instructed to report any adverse effects. Adverse effects reported by participants or documented by the physiotherapists during the treatment phases of the trial will be managed and reported (to ethics and relevant institutional unit) as per appropriate policies and procedures at the relevant site.

### Adverse events

Participants will be instructed to report any adverse events to the treating physiotherapists, and/or the trial investigators. Adverse effects reported by participants or treating physiotherapists during the treatment phases of the trial will be recorded, managed and reported (to ethics and relevant institutional unit) as per appropriate policies and procedures at the relevant site immediately. Appropriate follow up health and medical care will be recommended should it be required for any adverse event. All cases of adverse events will be followed up to ensure resolution.

## Discussion

The primary aim of this trial is to determine whether midfoot width mobility is a treatment effect modifier for foot orthoses compared to hip exercises. The ability to confidently predict a preferential response to any physical treatment for PFP, such as foot orthoses, has proven elusive to date and has at times been somewhat contentious [19, 80, 81]. Follow up analyses of previous work in our research unit [11, 18] on two different samples of participants with PFP has revealed that a reliable and easily administered measure of midfoot width mobility might predict those who will report a successful outcome after receiving foot orthoses. For example, a randomized clinical trial reported a success rate of 78% (7 of 9 cases) with foot orthoses in patients with PFP who had high midfoot width mobility compared to only a 20% success rate (2 of 10 cases) in those who had low midfoot width mobility [11]. Methods for defining a successful outcome and categorizing midfoot width mobility were similar to those being used in this current protocol. A successful outcome was unlikely to be related to natural history because only 5% (1/20) of the participants in the wait-and-see group had a successful outcome. A significant limitation of these data is that single group analyses were used [19]. Absence of a comparison group in the analysis means it is not possible to differentiate predictors of the general course of the condition regardless of treatment (i.e. prognostic factors) from predictors of outcome to a specific treatment (i.e. treatment effect modifiers) [16].

The design of the FOHX trial allows for robust testing of midfoot width mobility as a treatment effect modifier for foot orthoses compared to progressive resisted hip exercises in individuals who have PFP. It will first test if midfoot width mobility of ≥11 mm, which was defined on the basis of our previous work [11] will predict a preferential response to foot orthoses versus hip exercises. Given that this previous work was based on small sample sizes, we will also conduct a secondary analysis, in which midfoot width mobility will be treated as a continuous level measure to ensure that we have fully evaluated the hypothesis that midfoot width mobility is a treatment effect modifier. If the hypothesis is confirmed, then midfoot width mobility could help clinicians tailor treatment for patients who have PFP.

Apart from our previous research suggesting that midfoot width mobility may be predictive of a success following treatment with foot orthoses, there is *prima facie* evidence to support that foot orthoses will be more successful when the patient has a mobile foot. Distal to the knee, abnormal foot pronation has been hypothesised to induce adverse lower limb kinematic motions, which are associated with excessive load at the patellofemoral joint [82, 83]. Foot orthoses have a mechanical effect on foot pronation [84], so it is plausible that foot orthoses might have a mechanical effect on the patellofemoral joint [85–87]. Interestingly, a modeling study of foot orthoses on patellofemoral joint load indicated that while there was a significant effect, there was considerable inter-individual variation in the response [85] which further underpins the need to determine whether midfoot width mobility is a treatment effect modifier for foot orthoses. There is also a growing body of evidence that supports the efficacy of foot orthoses for people with PFP [10, 11, 88] but these clinical trials did not specifically examine if the foot orthoses were most useful in patients with mobile feet.

The head-to-head comparison between treatments that target regions distal and proximal to the patellofemoral joint has not been done, making the clinical trial outlined in this protocol novel. Proximal to the knee, neuromuscular dysfunctions at the hip and pelvis have been hypothesised to impact upon the patellofemoral joint kinematics [83, 89, 90]. Evidence suggests weakness of the postero-lateral hip musculature in primarily the hip abductor and external rotator muscle groups as a common impairment in those with PFP [67, 68, 91, 92]. Clinical trials that have compared isolated postero-lateral hip musculature exercises to no exercises or as part of a rehabilitative program have reported beneficial outcomes for patients who have PFP [12, 27–31]. This evidence supports exercises targeting the posterolateral hip musculature as a viable treatment option for those with PFP, and an appropriate comparator treatment option in this trial.

This trial protocol aims to minimise potential biases, optimise methodological quality and report pragmatic clinical findings by addressing key methodological limitations of previous studies that have aimed to investigate treatment effect modifiers for PFP. Key strengths of the trial include: (i) randomization of participants according to a schedule that will be generated by an independent body, (ii) enrollment based on pre-determined criteria by registered physiotherapists and independent of treatment allocation, (iii) participant stratification into pre-determined subgroups based on preliminary data, (iv) blinding of participants, assessors and therapists to critical information (e.g., trial hypothesis, stratification status, treatment allocation, baseline and follow-up outcome measures), (v) head-to-head comparison of two efficacious treatments for PFP, (vi) sufficiently powered sample size to detect a significant and substantial effect of midfoot width mobility as a treatment effect modifier for foot orthoses, (vii) blinded analysis using a pre-determined statistical analysis plan, and (viii) conducting a pre-specified secondary analysis to further evaluate midfoot width mobility as a treatment effect modifier for foot orthoses when it is a continuous variable. The findings from this trial will be reported in accordance to the CONSORT statement [21] and widely disseminated.

## Conclusion

In conclusion, this trial sets out to address two contentious issues that confront clinicians who treat patients with PFP. One looks to assist the clinician in determining who is likely to have a preferential response to foot orthoses treatment, compared to hip exercises, by testing if a simple, clinically applicable measurement of midfoot width mobility can be used to predict a better outcome. The second is to assist in optimising the management of PFP by comparing hip exercises to the use of foot orthoses.
